# Correlation of urine ammonia excretion with renal function in healthy dogs and dogs with chronic kidney disease

**DOI:** 10.1093/jvimsj/aalaf016

**Published:** 2026-01-21

**Authors:** Autumn N Harris, Alexis Copper, Rebeca A Castro, Andrew J Specht, Allison R Kendall, Shelly L Vaden, Kirsten L Cooke

**Affiliations:** Department of Clinical Sciences, North Carolina State University College of Veterinary Medicine, Raleigh, NC 27607, United States; Department of Small Animal Clinical Science, University of Florida College of Veterinary Medicine, Gainesville, FL 32608, United States; Department of Small Animal Clinical Science, University of Florida College of Veterinary Medicine, Gainesville, FL 32608, United States; Department of Clinical Sciences, North Carolina State University College of Veterinary Medicine, Raleigh, NC 27607, United States; Department of Small Animal Clinical Science, University of Florida College of Veterinary Medicine, Gainesville, FL 32608, United States; Department of Clinical Sciences, North Carolina State University College of Veterinary Medicine, Raleigh, NC 27607, United States; Department of Clinical Sciences, North Carolina State University College of Veterinary Medicine, Raleigh, NC 27607, United States; Department of Small Animal Clinical Science, University of Florida College of Veterinary Medicine, Gainesville, FL 32608, United States

**Keywords:** acid-base homeostasis, assays, canines, kidneys, renal physiology

## Abstract

**Background:**

Inadequate ammonia excretion is thought to drive the development of metabolic acidosis in people with chronic kidney disease (CKD) and correlates with worse clinical outcomes, such as faster progression to end-stage kidney disease and increased case fatality.

**Hypothesis/Objectives:**

To determine if urine ammonia-to-creatinine ratio (UACR) correlates with serum creatinine concentration as a renal function marker in healthy dogs and dogs with CKD and whether UACR is altered in the presence of CKD.

**Animals:**

The study group comprised 46 healthy and 50 stable International Renal Interest Society (IRIS) Stages 2, 3, and 4 CKD dogs.

**Methods:**

This was a prospective, single–time point study. Serum biochemistry variables were measured. Urinary ammonia and creatinine concentrations were measured and used to calculate UACR. Group comparisons were made with the Mann–Whitney test. Correlation between UACR and serum renal and electrolyte values was assessed using Spearman’s correlation test. Relationships between UACR, renal variables, electrolytes, urine specific gravity, age, and body weight were explored with multiple linear regression.

**Results:**

CKD dogs had lower UACR (median 2.2; range 0.9-10.5) than healthy dogs (median 7.1; range 0.7-40.0) (*P* < .001). UACR was inversely correlated with creatinine concentrations (*P* < .001, *r_s_* = −0.535). The relationship between UACR and creatinine persisted after controlling for age, body weight, electrolytes, renal functional variables, and urine specific gravity.

**Conclusions and clinical importance:**

These findings suggest that ammonia excretion is impaired in dogs with diminished renal function.

## Introduction

The kidneys play a central role in maintaining systemic acid-base homeostasis through 2 key mechanisms: reabsorption of filtered bicarbonate (HCO_3_^−^) and the generation of new bicarbonate to buffer endogenous acid production. Although complete reabsorption of filtered bicarbonate is essential, it is not sufficient to maintain acid-base balance on its own. To compensate for the continual daily acid load, the kidneys must generate new bicarbonate, a process that occurs through net acid excretion. Net acid excretion is the combined urinary elimination of titratable acids and ammonium (NH_4_^+^), with ammonia metabolism and subsequent ammonium excretion being the predominant contributor to net acid excretion under both basal conditions and in response to exogenous acid loads.^1^^,^^2^ Ammonia is predominantly derived from glutamine metabolism within the proximal tubules, resulting in the equimolar generation of NH_4_^+^ and new HCO_3_^−2^. Ammonia excretion in the protonated forms is the primary source of new bicarbonate generation.^1^^,^^2^

Chronic kidney disease (CKD) is a clinically relevant condition in aging dogs, with an estimated prevalence of 0.5% to 1.0% in the general canine population and increasing with age, posing a challenge due to its progressive nature and the difficulty of early detection.^3^ Identifying reliable markers and potential therapeutic targets in the early stages of CKD remains a critical need in veterinary medicine. One well-recognized complication of CKD is metabolic acidosis, with reported prevalence rates as high as 71% in affected dogs and approximately 40% in humans.^2^^,^^4^ Metabolic acidosis contributes to a range of detrimental clinical outcomes in humans, including protein-energy wasting, skeletal muscle catabolism, mineral and bone disorders, impaired cardiac function, and disruptions in glucose metabolism, all of which can negatively impact the quality of life.^5^^,^^6^ Impairment of ammonia excretion is a key contributor to the development of metabolic acidosis in humans with CKD.^6–8^ Studies of 24-hour urinary ammonia excretion in humans with CKD have demonstrated that reduced ammonia excretion is associated with worse clinical outcomes, including more rapid progression of CKD and death.^8^ In experimental models, the canine kidney has similarly been shown to metabolize glutamine to generate ammonia under basal conditions, with ammonia excretion increasing in response to acid loading.^9^ Although reference intervals for UACR have been previously established in a subset of healthy dogs,^10^ the mechanisms governing acid-base regulation in dogs with CKD, including the capacity for urinary ammonia excretion, remain poorly understood.

The objectives of this study were to determine if the urinary ammonia-to-creatinine ratio (UACR) correlated with serum creatinine concentrations as a renal function marker in a group of healthy dogs and dogs with CKD from a veterinary teaching hospital. A secondary objective was to determine whether UACR was related to the presence of CKD. We hypothesized that dogs with CKD would have a lower UACR than healthy dogs. We hypothesized that UACR would negatively correlate with serum creatinine concentrations and, therefore, be lower in the presence of CKD.

## Materials and methods

This was a prospective, observational study of healthy dogs and dogs with CKD that presented to the internal medicine service or the primary care service at the University of Florida or the nephrology urology service at North Carolina State University. This study was approved by the University of Florida and North Carolina State University Institutional Animal Care and Use Committee (202111549; 24-308) and written consent was obtained from owners.

### Study cohorts

The apparently healthy dog group samples were collected from dogs that presented at the University of Florida primary care service for wellness examination. These dogs were placed in the healthy category based on the absence of findings suggesting clinically relevant illness, as indicated by the medical history, physical examination findings, serum biochemistry, and urinalysis. Dogs were excluded from this category if they had azotemia or had been previously diagnosed with another disease likely to alter renal function, if urine samples had cytologic evidence of inflammation (≥ 5 white blood cells [WBC]/high-powered field) or evidence of bacteriuria, evidence of clinically relevant dipstick proteinuria (> trace), or if they received glucocorticoids, diuretics, urinary acidifiers, alkalinizing agents, ARB (angiotensin-receptor blockers), or ACE (angiotensin-converting enzyme) inhibitors. Other medications were permitted if they did not have any known association with renal function or ammonia metabolism.

For the kidney disease group, client-owned dogs greater than 1 year of age that presented to the internal medicine service at the University of Florida Small Animal Hospital or the nephrology urology service at North Carolina State University Small Animal Hospital for medical treatment or monitoring of CKD International Renal Interest Society (IRIS) Stage 2 or greater were enrolled in this study. Dogs were eligible for inclusion in the CKD group if they met the following criteria on at least 2 visits, separated by a minimum of 2 weeks and maximum of 6 months: a serum creatinine concentration > 1.4 mg/dL or serum symmetric dimethylarginine concentration (SDMA) > 18 μg/dL, lack of appropriate urine concentration (urine specific gravity < 1.035), and stable azotemia (<25% change between measurements). All eligible dogs also had to be eating the same therapeutic renal diet with no changes for at least 28 days, and be well hydrated based on physical examination and serial body weight measurements. Health status was assessed through a comprehensive physical examination and complete staging of their CKD, including Doppler systolic blood pressure measurement, a biochemistry panel, packed cell volume/total protein (PCV/TP), urinalysis, and a urine protein-to-creatinine ratio (UPC). Dogs were excluded if they had an active urine sediment, defined as having greater than 5 WBC/high-power field, greater than 5 red blood cells/high-power field, or bacteriuria, if they had comorbidities that might alter glomerular filtration rate (GFR), or if they required hospitalization for CKD management or were receiving alkali therapy or potassium-sparing diuretics.

### Sample collection and storage

Samples from each dog were acquired during a single hospital visit at the time of study enrollment. All dogs had food withheld for 8 to 10 hours before sample collection. At least 5 mL of urine was obtained via free catch or cystocentesis. After collection, 0.5 to 1.0 mL of urine was immediately placed into a sterile conical tube with 1 mL of mineral oil to prevent evaporation. The samples were processed by placing urine into cryotubes within 4 hours of initial collection and stored at −80°C for no longer than 6 months. Samples were then batched for analysis.

### Analytical methods

Urinalyses were performed by the University of Florida or North Carolina State University clinical pathology laboratory on the same day as the dog’s hospital visit. A refractometer was used to assess urine specific gravity (USG), and a semiquantitative dipstick analysis was performed by visual inspection. Microscopic evaluation of urine sediment was performed to screen for components of active sediment, including red blood cells, white blood cells, or bacteria.

Serum biochemistry panels were performed through the clinical pathology laboratories at University of Florida and North Carolina State University. Frozen urine samples were thawed and then centrifuged at 2,100 *g* for 2 minutes (Thermo Scientific Sorvall Legend micro21R) with 400 μL of supernatant removed to measure ammonia and creatinine. A commercially available enzymatic assay (Ammonia Reagent Assay; Pointe Scientific, Canton, MI) was used to measure urine ammonia concentrations. Urine creatinine concentrations were determined with the modified Jaffe method using a commercially available assay (Creatinine Assay Kit, ab204537; Abcam, Cambridge, MA). The coefficient of variation and total observed error for both assays have been previously reported.^11^ A SpectraMax ABS plus microplate reader (Molecular Devices) was used for ammonia (mmol/L) and creatinine (mg/dL) measurements. To keep units consistent for ammonia and creatinine, measures for the latter were converted from mg/dL to mmol/L by multiplying by 0.055. Ammonia-to-creatinine ratio (UACR) was then calculated accordingly:

$UACR=\displaystyle\frac{\mathrm{urine}\ \mathrm{ammonia}\ \mathrm{concentration}}{\mathrm{urine}\ \mathrm{creatinine}\ \mathrm{concentration}}$ .

### Data collection

Demographic data, including age, breed, sex, and weight, were recorded. The serum chemistry panel results were recorded, and dogs were classified as having metabolic acidosis if their bicarbonate concentration was ≤ 18 mEq/L. For the CKD dogs, stage and substages (based on IRIS guidelines for serum creatinine concentrations, systolic blood pressure, and UPC) were recorded.

### Statistical analysis

The data were compiled in an Excel spreadsheet (Microsoft Office, 2019) and statistically analyzed with commercially available software (Graph Pad Prism version 10). The sample size was determined a priori by estimating the number of cases needed to demonstrate a relationship with *r* = 0.3, significance *P* < .05, and power of 80%, assuming normality of data, absence of extreme observations, and a plausibly linear relationship between variables.^12^ Clinicopathologic data were tested for normality using the Shapiro–Wilk test and presented as mean (± SD) if normally distributed or as the median (range) if non-normally distributed. Descriptive statistics were used to summarize all variables, including UACR; signalment data (age, breed, sex, and weight); systolic blood pressure; and laboratory data (serum bicarbonate, creatinine, BUN, phosphate, potassium, chloride, PCV, and UPC). An unpaired Student *t* test or Mann–Whitney test was performed depending on normality testing to compare demographic and clinicopathologic variables between the healthy and CKD groups. A Chi-squared test was used to compare the proportions of sex in each group. Statistical significance was set to *P* < .05. The correlation between UACR and serum renal and electrolyte concentrations were explored with Spearman’s correlation test. A Bonferroni’s correction was applied, indicating that an adjusted *P* < .005 was appropriate to minimize the risk of false-positive results. Multilinear regression was performed to examine the effect of serum creatinine concentrations and other independent variables identified through correlation on UACR. The response variables for the model were UACR and explanatory variables included serum creatinine, BUN, phosphate, calcium, potassium, chloride, bicarbonate, USG, age, and body weight. Significance was set at *P* < .05.

## Results

Forty-six healthy dogs and 50 dogs with CKD were included in this study. Eleven dogs were excluded from the healthy group for various reasons, including renal azotemia (*n* = 4) and elevated serum activity of liver enzymes (*n* = 7). No dogs were excluded from the CKD group. The healthy group of dogs included 20 neutered males, 23 spayed females, 1 intact female, and 2 intact males. Mixed-breed dogs comprised the majority of the study samples (*n* = 18). Purebred dogs included American Staffordshire terrier (*n* = 5), golden retriever (*n* = 3), Akita (*n* = 2), Greyhound (*n* = 2), Great Dane (*n* = 2), Labrador retriever (*n* = 2), Bassett hound (*n* = 2), and 1 of each of Springer spaniel, goldendoodle, Kerry Blue terrier, English bulldog, Jack Russell terrier, Doberman pinscher, German shepherd, Black Mouth cur, American dingo, and Dachshund. Ages ranged from 1 to 15 years (median 7 years). Body weights ranged from 7.1 to 56.0 kg (median 27.6 kg).

The CKD group included 27 neutered males, 19 spayed females, 3 intact females, and 1 intact male. The CKD group included the following breeds: mixed-breed (*n* = 13), Yorkshire terrier (*n* = 5), pit bull (*n* = 4), Labrador retriever (*n* = 3), Chihuahua (*n* = 3), Australian shepherd (*n* = 3), Welsh corgi (*n* = 2), beagle (*n* = 2), and 1 of the following: Nova Scotia duck tolling retriever, Doberman pinscher, Great Dane, Cavalier King Charles spaniel, English bulldog, goldendoodle, Jack Russell terrier, Irish setter, Maltese, wheaten terrier, golden retriever, Boykin spaniel, Dalmatian, Rhodesian ridgeback, and border collie. Ages ranged from 1 to 17 years (median 12 years). Body weight ranged from 1.8 to 67.0 kgs (median 15.1 kg). Forty-one dogs (82%) were classified as IRIS Stage 2; 7 dogs (14%) were classified as IRIS Stage 3; and 2 dogs (4%) were classified as IRIS Stage 4. Additional substaging information is included in Supplemental Table 1. All CKD dogs were fed a therapeutic renal diet with a protein range of 3.4 to 4.2 g/100 kcal. Eighteen (36%) were not receiving any medications, and 32 (64%) were receiving medications at the time of enrollment, which included amlodipine (*n* = 8), aluminum hydroxide (*n* = 7), trazadone (*n* = 5), gabapentin (*n* = 5), phenylpropanolamine (*n* = 4), polysulfated glycosaminoglycan (*n* = 3), telmisartan (*n* = 3), tramadol (*n* = 3), maropitant citrate (*n* = 3), pimobendan (*n* = 3), carprofen (*n* = 3), galliprant (*n* = 2), clopidogrel (*n* = 2), cisapride (*n* = 2), ondansetron (*n* = 2), famotidine (*n* = 2), omeprazole (*n* = 1), Dasuquin (*n* = 1), selegiline (*n* = 1), enalapril (*n* = 1), tamsulosin (*n* = 1), mirtazapine (*n* = 1), and capromorelin (*n* = 1).

**Figure 1:**
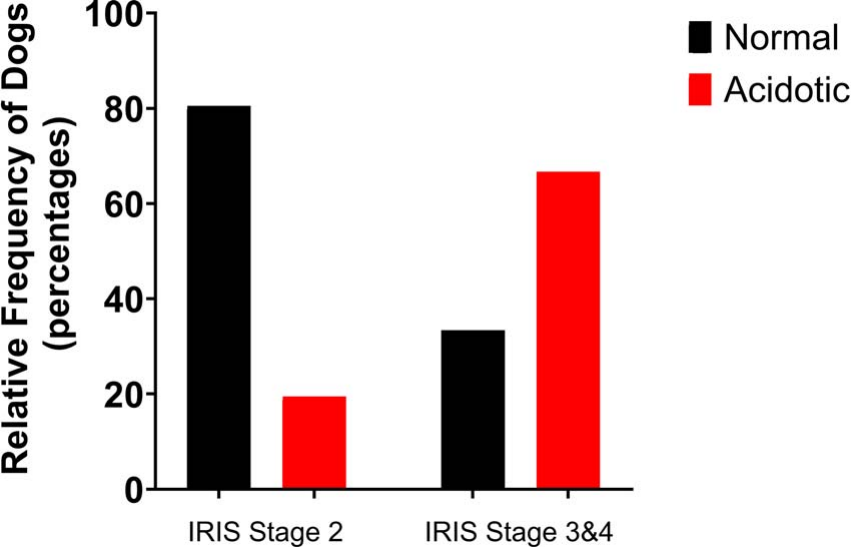
Distribution of metabolic acidosis based on serum bicarbonate concentrations in dogs with CKD at different stages of their disease. Early CKD (International Renal Interest Society [IRIS] Stage 2), *n* = 41; late CKD (IRIS Stages 3 and 4), *n* = 9. Abbreviation: CKD = chronic kidney disease.

**Figure 2:**
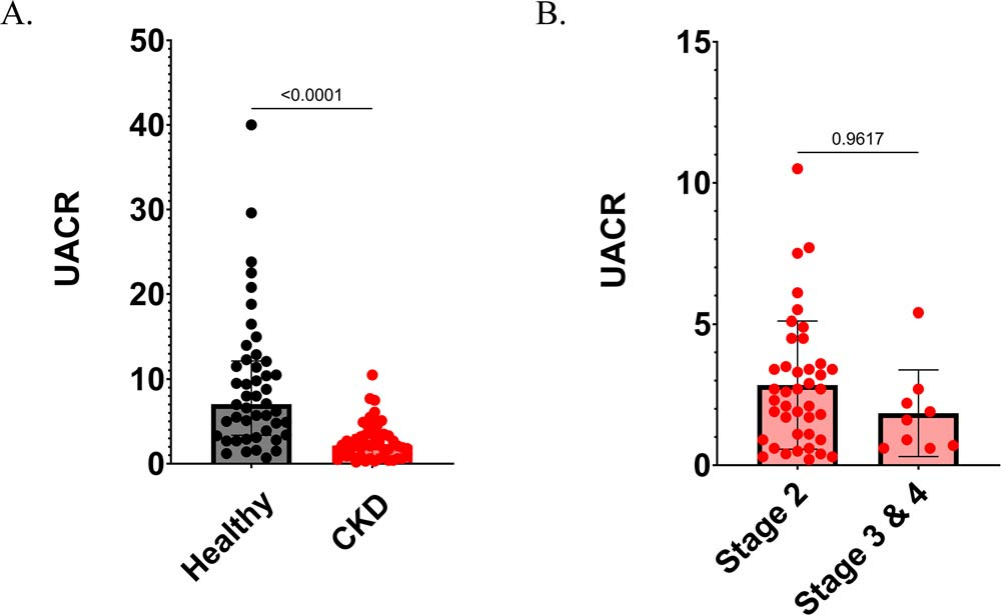
(A) Comparison of urine ammonia-to-creatinine ratio (UACR) between healthy and chronic kidney disease (CKD) groups. The center line denotes the median value (50th percentile), the box contains the 25th to 75th percentiles, the whiskers mark the 5th and 95th percentiles. UACR is significantly lower in CKD dogs compared to healthy dogs (*P* < .0001). Healthy, *n* = 46; CKD, *n* = 50. (B) Comparison of UACR between early CKD (International Renal Interest Society [IRIS] Stage 2; *n* = 41) and late CKD (IRIS Stages 3 and 4; *n* = 9) groups. The center line denotes the median value (50th percentile), the box contains the 25th to 75th percentiles, the whiskers mark the 5th and 95th percentiles. There is no significant difference in UACR between early-stage and late-stage CKD dogs.

Demographic and clinicopathologic variables are presented in Table 1 for comparison between healthy and CKD dogs. Healthy dogs were significantly younger and heavier than dogs with CKD. Serum calcium and potassium concentrations were significantly higher, whereas serum bicarbonate and chloride concentrations were significantly lower, in dogs with CKD compared to healthy dogs. There was no significant difference in serum phosphate concentrations between healthy dogs and dogs with kidney disease. The overall prevalence of metabolic acidosis in healthy dogs based on serum bicarbonate concentrations was 4%, and in CKD dogs it was 30%, including 21% of dogs with early CKD (IRIS Stage 2) and 67% of dogs with late-stage CKD (IRIS Stages 3 and 4; Figure 1). There was a significant difference in UACR between healthy and CKD dogs, with the CKD dogs having a significantly lower UACR (Table 1; Figure 2). In the dogs with CKD there was no significant difference in UACR between dogs with early kidney disease (IRIS Stage 2) versus those with late-stage kidney disease (IRIS Stages 3 and 4; Figure 2).

**Table 1:** Demographic and clinicopathologic variable comparisons between healthy dogs and dogs with chronic kidney disease.

**Variable**	**Healthy (*n* = 46)**	**CKD (*n* = 50)**	***P*-value**
**Age, months**	81 (22-184)	146 (12-199)	**<.0001**
**Body weight, kg**	27.6 (7.1-56.0)	15.1 (1.8-67.0)	**<.0001**
**Sex, *n***	20 MN, 23 FS, 1 FI, 2 MI	27 MN, 19 FS, 3 FI, 1 MI	.34
**UACR**	7.1 (0.7-40.0)	2.2 (0.9-10.5)	**<.0001**
**Serum creatinine, mg/dL**	1.0 (0.5-1.2)	2.0 (0.9-5.8)	
**Serum BUN, mg/dL**	19 (10-43)	34 (6-144)	
**Serum phosphate, mg/dL**	3.7 (2.0-5.0)	4.0 (2.2-8.1)	.135
**Serum calcium, mg/dL**	9.7 (8.7-10.5)	10.8 (9.0-12.3)	**<.0001**
**Serum potassium, mEq/L**	4.3 (3.6-5.2)	4.8 (3.3-6.1)	**<.0001**
**Serum chloride, mEq/L**	113.1 (108.3-119.2)	111.7 (102.5-118.8)	** .02**
**Serum bicarbonate, mEq/L**	22 (16-26)	20 (12-28)	** .03**
**Urine specific gravity**	1.032 (1.006-1.056)	1.014 (1.000-1.030)	

Data presented as median (range). Significant differences at *P* < .05 are bolded. Reference ranges for laboratory values: creatinine 0.5-1.4 mg/dL; BUN 11-43 mg/dL; phosphate 2.6-5.3 mg/dL; calcium 9.5-11.1 mg/dL; potassium 3.6-5.3 mEq/L; chloride 107-115; bicarbonate 18-25 mEq/L. Abbreviations: BUN = blood urea nitrogen; CKD = chronic kidney disease; FI = female intact; FS = female spayed; MI = male intact; MN = male neutered; UACR = urine ammonia-to-creatinine ratio.

**Figure 3:**
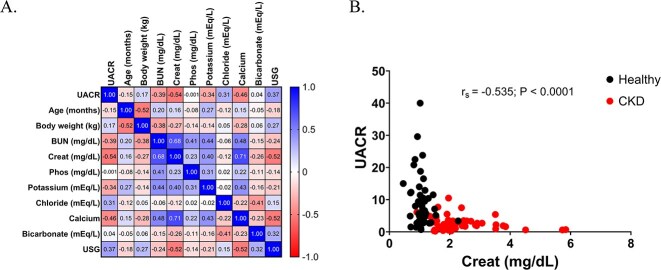
(A) Spearman’s correlation heat map shows the relationships between measured variables in all dogs in this study. Positive correlation coefficients are depicted in blue and negative correlation coefficients are depicted in red. (B) Significant and moderately negative correlation between urine ammonia-to-creatinine ratio and serum creatinine is shown for all 96 dogs enrolled in this study, including healthy dogs (*n* = 46, represented by black circles), and those with chronic kidney disease (*n* = 50, represented by red circles) (*P* < .0001; *r_s_* = −0.535). Abbreviations: BUN = blood urea nitrogen; Cl^−^ = chloride concentration; HCO_3_^−^ = bicarbonate concentration; K^+^ = potassium concentration; UACR = urine ammonia to creatinine ratio; USG = urine specific gravity.

The UACR was negatively correlated with serum creatinine, BUN, potassium, and calcium concentrations and was positively correlated with serum chloride concentrations and USG (Table 2, Figure 3). There was no significant correlation between UACR and serum phosphate and bicarbonate concentrations (Table 2; Figure 3). Body weight, serum creatinine, phosphate, and chloride concentrations remained significantly associated with UACR after controlling serum BUN, calcium, potassium, bicarbonate, USG, and age (Table 3).

**Table 2:** Correlation between urine ammonia-to-creatinine ratio and measured variables for all dogs (*n* = 96).

**Variable 1**	**Variable 2**	***r***_***s***_	**95% CI**	***P*-value**
**UACR**	Age	−0.150	−0.35 to −0.06	.144
**UACR**	Body weight	0.167	−0.04 to 0.36	.103
**UACR**	Creatinine	−0.535	−0.67 to −0.37	**<.0001**
**UACR**	BUN	−0.392	−0.55 to −0.20	**<.0001**
**UACR**	Phosphate	−0.001	−0.21 to 0.21	.993
**UACR**	K^+^	−0.345	−0.51 to −0.15	** .001**
**UACR**	Cl^−^	0.307	0.11 to 0.48	**.002**
**UACR**	Calcium	−0.464	−0.62 to −0.29	**<.0001**
**UACR**	HCO_3_^−^	0.041	−0.17 to 0.25	.689
**UACR**	USG	0.372	0.18 to 0.54	**<.0001**

Statistically significant differences at the adjusted *P* < .005 are bolded. The correlation coefficient *r_s_* and 95% CIs are shown. Abbreviations: BUN = blood urea nitrogen; Cl^−^ = chloride concentration; HCO_3_^−^ = bicarbonate concentration; K^+^ = potassium concentration; UACR = urine ammonia-to-creatinine ratio; USG = urine specific gravity.

**Table 3:** Multiple linear regression model with UACR as response variable.

**Exploratory variables**	**β coefficient**	**95% CI**	**Adjusted *P*-value**
**Age**	0.017	−0.011 to 0.046	.248
**Body weight**	0.138	0.021 to 0.255	** .022**
**BUN**	−0.016	−0.101 to 0.069	.695
**Creatinine**	−2.740	−5.205 to −0.640	** .013**
**Phosphorus**	2.189	0.727 to 3.606	** .003**
**K** ^**+**^	−2.166	−4.830 to 0.544	.111
**Cl** ^**−**^	0.504	0.003 to 0.984	** .043**
**Calcium**	0.929	−1.266 to 3.275	.412
**HCO**_**3**_^**−**^	0.332	−0.170 to 0.835	.201
**USG**	58.32	−57.27 to 172.2	.312

The overall P value for the model was <.0001. The *R^2^* for the model was 0.3180. Statistically significant differences are bolded. The β coefficient and 95% CI are shown. Abbreviations: BUN = blood urea nitrogen; Cl^−^ = chloride concentration; HCO_3_^−^ = bicarbonate concentration; K^+^ = potassium concentration; UACR = urine ammonia-to-creatinine ratio; USG = urine specific gravity.

## Discussion

A major finding of this study is the moderate inverse correlation between urinary ammonia excretion and renal function, as assessed by serum creatinine concentration. This relationship mirrors findings in human medicine, in which net acid excretion, primarily driven by ammonium excretion, declines with decreasing glomerular filtration rate (GFR) and is independently associated with worse clinical outcomes.^7^^,^^8^^,^^13^ A similar trend exists in cats with CKD.^14^ The reduction in ammonium excretion with progressive kidney dysfunction is likely multifactorial. Proposed mechanisms include a reduced number of functioning nephrons, impaired uptake of glutamine by proximal tubular cells (a key substrate for renal ammonia production), and a diminished NH₃ concentration gradient within the renal interstitium, which limits effective ammonia secretion into the tubular lumen.

The moderate inverse relationship between ammonia excretion and renal function (as indicated by serum creatinine concentration) suggests that factors other than serum creatinine may contribute to ammonia excretion. This is an expected result because serum creatinine is predominantly a surrogate for GFR as a reflection of renal function, whereas ammonia excretion is most likely directly related to tubular function. Because GFR is not a perfect reflection of renal tubular function, serum creatinine would likely not be perfectly correlated with ammonia excretion. Despite this, serum creatinine concentrations remained significantly associated with ammonia excretion after controlling for independent variables. Notably, body weight, serum phosphate, and chloride concentrations were also significantly correlated with ammonia excretion. Chloride plays a well-established role in maintaining acid-base balance, and as expected it was related to ammonia excretion. Chloride may reflect the body’s compensatory response to retained hydrogen ions due to impaired acid excretion.^15^^,^^16^ Additionally, chloride reabsorption in the proximal tubule is tightly linked to sodium and bicarbonate handling, and may indirectly influence ammonia production and transport. The relationship between phosphate and ammonia excretion is likely multifactorial. In CKD, phosphate retention occurs due to lower renal excretion, which worsens with progressive nephron loss.^17^^,^^18^ An inverse relationship between serum phosphate and ammonia excretion could indicate progressive tubular dysfunction, with which phosphate retention and lower ammonia generation occur in parallel. Finally, body weight may influence ammonia excretion in several ways, particularly through its effects on metabolic demands and acid-base regulation. Ammonia is primarily produced from glutamine metabolism in the kidneys, and muscle tissue serves as a key source of glutamine.^2^ Given that body weight is often correlated with lean body mass (muscle mass), a dog with lower body weight may have less muscle available to produce glutamine, resulting in less ammonia generation and excretion.

This is the first study to demonstrate that dogs with kidney disease exhibit significantly impaired ammonia excretion compared to healthy dogs. Notably, this impairment was observed in both early-stage and late-stage CKD, suggesting that disruption of renal ammonia handling begins early in the disease process. These findings parallel recent observations in cats with CKD, in which reduced ammonia excretion was also evident in early stages.^14^ Impaired ammonia excretion may contribute to a positive acid balance and renal injury before detectable serum bicarbonate concentrations change. However, further research is needed to determine whether ammonia excretion in dogs with CKD has prognostic significance or could serve as a therapeutic target in clinical management.

Although urinary ammonia excretion contributes to the generation of new bicarbonate in response to systemic acid loads, our study did not find a significant correlation between UACR and serum bicarbonate concentration. This finding is consistent with prior literature suggesting that serum bicarbonate does not always reflect renal acid excretory capacity, particularly in early or subclinical stages of renal dysfunction.^7^^,^^19^ Several physiological mechanisms may explain this disconnect. First, extrarenal buffering systems, including bone, intracellular proteins, and muscle, can temporarily maintain serum bicarbonate despite impaired urinary ammonia excretion.^5^ Second, differences in dietary acid load, gastrointestinal alkali loss, and tissue acid production can independently influence bicarbonate levels, apart from renal ammonia handling.^20^ Third, renal ammonia production and excretion may be blunted in CKD or tubular dysfunction even before serum bicarbonate concentrations begin to fall, particularly when nephron mass is reduced.^8^ Therefore, although UACR reflects a component of renal acid excretion, serum bicarbonate integrates multiple systemic processes and may not track urinary ammonia excretion linearly under all circumstances.

A presumptive diagnosis of metabolic acidosis is usually made in dogs with CKD when serum bicarbonate concentrations are consistently below the reference range. This is because blood gases are not readily available in many outpatient settings and are unnecessary in most cases. In this study, dogs with kidney disease had significantly lower serum bicarbonate concentrations than healthy dogs. Evidence of metabolic acidosis, defined as a serum bicarbonate concentration <18 mmol/L, was present in 30% of CKD dogs, comparable to a recent study reporting a prevalence of 42%.^4^ As expected, the prevalence of acidosis increased with CKD stage, occurring in 21% of dogs with early-stage disease (IRIS Stage 2) and rising to 67% in those with late-stage CKD (Stages 3 and 4). These findings suggest that metabolic acidosis may be more common in dogs with early-stage CKD than previously appreciated.

Although the prevalence of metabolic acidosis increased across IRIS CKD stages, we did not observe a statistically significant difference in UACR between groups. This apparent discordance may be attributed to several physiological and methodological factors. First, UACR, although applicable as a snapshot of ammonia excretion relative to renal filtration, may not fully reflect total ammonia output, especially in advanced CKD, in which creatinine production diminishes with muscle mass and GFR.^5^^,^^21^ Second, tubulointerstitial injury associated with CKD progression may selectively impair ammonia production and secretion, even before measurable changes in GFR or creatinine clearance are evident.^22^ Third, the development of acidosis in advanced CKD likely reflects not only a decline in ammonia excretion but also a reduced buffer reserve, increased dietary acid load, and loss of skeletal buffering capacity.^23^^,^^24^ Therefore, the presence of acidosis in later CKD stages without a clear gradient in UACR underscores the complex interplay between acid load, renal adaptive mechanisms, and extrarenal compensation in the regulation of systemic pH.

Several limitations of this study should be considered when interpreting the results. First, the temporal and biological variability of urinary ammonia-to-creatinine ratios (UACR) in dogs, both in health and disease, has not been previously characterized. Although samples in this study were collected during clinical appointments and after food was withheld, variability in collection times may have influenced the results. Second, efforts to exclude comorbid conditions in the healthy group were based on history, physical examination, and limited laboratory testing. Comprehensive evaluations were not performed, such as GFR measurement, renal biopsies, or advanced hematologic testing. Therefore, the possibility that some dogs classified as healthy had subclinical disease cannot be excluded, although efforts were made to minimize this risk. Third, although none of the dogs in the CKD group had clinical signs or urinalysis findings suggestive of urinary tract infection, subclinical bacteriuria cannot be definitively ruled out, as urine cultures were not performed. Last, the number of dogs with late-stage CKD (IRIS Stages 3 and 4) was relatively small, which may have limited the ability to detect statistically significant differences in ammonia excretion between early-stage and late-stage disease.

### Conclusion

These findings suggest that ammonia excretion becomes impaired with diminishing renal function, and that these changes occur early in dogs with CKD. Acidosis based on serum bicarbonate concentrations is common in dogs with CKD, occurring early in dogs with kidney disease and increasing in prevalence with advancing stage of disease.

## Supplementary Material

aalaf016_supplemental_table_1

## Off-label antimicrobial declaration

Authors declare no off-label use of antimicrobials.

## Institutional animal care and use committee or other approval declaration

The study was approved by the University of Florida (202111549) and North Carolina State University (24-308). Informed consent was obtained from the owners or legal custodians of all animals described in this work.

## Abbreviations

BUN: blood urea nitrogen
Ca^2+^: calcium
CKD: chronic kidney disease
Cl^−^: chloride
GFR: glomerular filtration rate
HCO_3_^−^: bicarbonate
K^+^: potassium
Na^+^: sodium
UACR: urine ammonia-to-creatinine ratio
UPC: urine protein-to-creatinine ratio
USG: urine specific gravity
