# Supplementary material for: Correlation of urine ammonia excretion with renal function in healthy dogs and dogs with chronic kidney disease
Source: J Vet Intern Med. 2026 Jan 21;40(1):aalaf016. doi: 10.1093/jvimsj/aalaf016 (PMC12881969; doi:10.1093/jvimsj/aalaf016)
Supplement: aalaf016_supplemental_table_1 [file aalaf016_supplemental_table_1.docx]

Supplemental Table 1. IRIS CKD stages and substages for CKD dog population.

| Stage 2 (n=41) | Non-proteinuric | Borderline proteinuric | Proteinuric |
| --- | --- | --- | --- |
| Not hypertensive | 3 | 2 | 5 |
| Pre-hypertensive | 5 | 3 | 4 |
| Hypertensive | 1 | 1 | 4 |
| Severely hypertensive | 7 | 3 | 3 |
|  |  |  |  |
| Stage 3 (n = 7) | Non-proteinuric | Borderline proteinuric | Proteinuric |
| Not hypertensive |  |  | 1 |
| Pre-hypertensive | 1 | 2 | 1 |
| Hypertensive |  |  |  |
| Severely hypertensive |  |  | 2 |
|  |  |  |  |
| Stage 4 (n = 2) | Non-proteinuric | Borderline proteinuric | Proteinuric |
| Not hypertensive |  |  |  |
| Pre-hypertensive |  |  | 1 |
| Hypertensive |  |  |  |
| Severely hypertensive |  |  | 1 |
